# The Fundamental Biological Factors Underlying the Incidence of Tuberculosis

**Published:** 1922-02

**Authors:** R. J. Ewart

**Affiliations:** (Medical Officer of Health and Tuberculosis Officer, Barking Town, E.)


### THE FUNDAMENTAL BIOLOGICAL FACTORS UNDERLYING THE INCIDENCE OF TUBERCULOSIS.

**By R. J. EWART, M.D., D.Sc., F.R.C.S. (Medical Officer of Health and Tuberculosis Officer, Barking Town, E.)**

THE factors influencing the rise and fall of an epidemic disease may be represented in the following way. The essential feature by which its presence is noted is the discomfort it causes to the people concerned. I suggest, therefore, that our attention should be mainly centred on them, and not on the agent or parasite.

- Germ plasm.

  - Preconceptional.

    - Inherent.

    - Pre-natal environment.

  - Post-conceptional.

    - Pre-natal maternal nutrition.

    - Post-natal environment.

      - Food.

      - Air.

    - Infection.

      - Variety.

      - Virulence.

        - Inherent.

        - Environment.

The scheme gives the following headings for consideration :—

(1) Inherent properties of living material.
(2) Preconceptional environment.
(3) Maternal nutrition.
(4) Food.
(5) Air.
(6) Variety of infecting agent.
(7) Virulence of infecting agent.

The disease taken to exemplify the above is Tuberculosis.

The reason why this disease has been selected is because of its relatively slow growth, which enables us to examine conflicting causes with more certainty than with the more explosive diseases.

The material that has been used was collected in the township of Barking, situated to the east of London, in the County of Essex. I have had every opportunity of forming an accurate idea of the relationship of the population to the parasite. As my office includes Medical Officer of Health, School Medical Officer, Tuberculosis Officer and Dispensary Officer, the information obtained should be fairly comprehensive.

The statistics relative to this condition, as far as the local records allow, are given in the table following.

It expresses the actual toll taken by the disease, and also the number dying had the population consisted of 1,000 persons. In the last column is given the chance of any individual terminating his life through the disease. This figure expresses the number of deaths from Pulmonary Tuberculosis per 100 deaths. It is seen that the chance of an inhabitant dying from this condition has increased from one in twenty to almost one in ten.

Coming to more modern times, and the use of tuberculin reactions, the statement may be accepted that the whole population has been infected by the twelfth to the fourteenth year, and has reacted to the presence of the parasite. In most the damage is nil, but in others according to their inherited constitution, the secondary changes are small or great in amount.

It has been suggested that the increase in Tuberculosis experienced in the area during the last 25 years is due to immigration from East London, and is dependent upon the desire of these families which are reacting badly to live in a more open neighbourhood, where rents and living are relatively cheap and transport charges are not excessive. Barking seems to fulfil these desiderata. Actual inquiry as to the place of former residence lends support to this view, as about 25 per cent. of the population are not born in the area. (East London, 21 per cent.) Whether this cause explains the whole of the rise observed cannot be definitely decided.

The relative accuracy of the diagnosis made is given in the following figures, taken from the post-mortem findings of a large general hospital.

Correct in 32 cases, or 59 per cent.

Error of omission, 16 cases, or 30 per cent.

Error of commission, 6 cases, or 11 per cent.

DEATHS FROM TUBERCULOSIS.
(All forms.)

| Year. | No. of deaths from Phthisis. | Rate per 1,000 pop. | Other forms of Tuberculosis. | Rate per 1,000 pop. | Percentage of deaths from Phthisis to total deaths, all causes. |
|---|---|---|---|---|---|
| 1897 | 20 | 1·02 (1·3) | ... | ... | 5·5 |
| 1898 | 11 | 0·54 (1·3) | ... | ... | 3·3 |
| 1899 | 16 | 0·75 (1·3) | ... | ... | 4·1 |
| 1900 | 24 | 1·1 (1·3) | 9 | 0·41 | 6·0 |
| 1901 | 22 | 1·0 (1·3) | 28 | 1·37 | 5·7 |
| 1902 | 21 | 0·91 (1·2) | 13 | 0·56 | 5·7 |
| 1903 | ... | — (1·2) | ... | ... | ... |
| 1904 | 26 | 1·05 (1·2) | 7 | 0·28 | 10·5 |
| 1905 | 22 | 0·85 (1·1) | 9 | 0·35 | 8·5 |
| 1906 | 20 | 0·75 (1·16) | 18 | 0·67 | 7·5 |
| 1907 | 39 | 1·41 (1·15) | 14 | 0·50 | 14·2 |
| 1908 | 36 | 1·26 (1·12) | 13 | 0·45 | 12·6 |
| 1909 | 36 | 1·22 (1·09) | 22 | 0·74 | 12·2 |
| 1910 | 25 | 0·82 (1·02) | 11 | 0·36 | 7·7 |
| 1911 | 40 | 1·26 (1·06) | 6 | 0·19 | 7·9 |
| 1912 | 28 | 0·86 (1·02) | 7 | 0·21 | 8·6 |
| 1913 | 50 | 1·49 (0·99) | 13 | 0·39 | 15·9 |
| 1914 | 48 | 1·38 (1·01) | 7 | 0·20 | 13·8 |
| 1915 | 54 | 1·56 (1·13) | 33 | 0·95 | 15·7 |
| 1916 | 44 | 1·29 (1·20) | 11 | 0·32 | 13·0 |
| 1917 | 74 | 2·04 (1·23) | 8 | 0·22 | 20·5 |
| 1918 | 55 | 1·55 (1·32) | 7 | 0·19 | 15·6 |
| 1919 | 35 | 0·99 (0·97) | 14 | 0·39 | 10·0 |
| 1920 | 38 | 1·99 (—) | 11 | 0·31 | 8·7 |

Figures in brackets—Death-rate of Phthisis per 1,000 living for England and Wales.

**The Fundamental Biological Factors Underlying the Incidence of Tuberculosis—(*cont.*).**

Taking figures from St. George's Hospital as far back as 1840, out of 2,101 post-mortem examinations, evidence of tuberculosis was found in :—

Birth to 15 years, 27·9 per cent.
15 to 30 years, 35·0 per cent.
30 to 45 years, 25·0 per cent.
46 to 60 years, 7·7 per cent.
All, 26·1 per cent.

Cummins, in the "National Journal of Public Health" (Vol. I., No. 2, September, page 143), gives some interesting figures relative to the reaction of isolated populations to Tuberculosis as judged by the tuberculin reaction :—

| | |
|---|---|
| French Guinea .. .. .. | 12 per cent. |
| German East Africa .. .. | 20 ,, |
| Annam .. .. .. .. | 48 ,, |
| Tartars (Central) .. .. | 52 ,, |
| ,, (Peripheral) .. .. | 82 ,, |
| Paris and Lille .. .. .. | 85 ,, |

The differences cannot be due to susceptibility, as the death rate from Phthisis of these people when they reside in European countries is extremely high.

Tuberculosis.

| | 1917. | | 1918. | |
|---|---|---|---|---|
| | Cases. | Deaths. | Cases. | Deaths |
| British troops ... ... | 1,660 | 91 | 1,221 | 74 |
| Cape boys ... ... ... | 156 | 69 | 171 | 113 |

Borril (quoted by above) brings out the same points in dealing with the French units.

Thus, the coloured divisions in France in 1916 showed a tuberculous evidence of 27·4 per 1,000, as compared with a case incidence of 1·1 per 1,000 amongst British troops.

These observations give decided support to the significance of the reaction to tuberculin, being a measure of the changes consequent upon infection by the bacillus.

*Percentage Showing Evidence of Infection by Tubercle Bacillus or Such as Have Acquired an Intolerance to its Presence.

| | By cutaneous reaction. | By cutaneous. reaction and injection. |
|---|---|---|
| 2nd year of life ... ... | 2 per cent. | 9 per cent. |
| 3rd to 4th year ... ... | 13 ,, | 27 ,, |
| 5th to 6th year ... ... | 17 ,, | 51 ,, |
| 7th to 10th year ... ... | 35 ,, | 71 ,, |
| 11th to 14th year ... ... | 55 ,, | 94 ,, |

From these data, it seems that Tuberculosis is much more common than is usually believed. In ordinary everyday life children experience periods of indifferent health and vigour, which is disturbing to the parent and unsatisfactory to the medical attendant, as little or no abnormality can be detected to account for the condition ; much of this must be due to unrecognisable tuberculosis and depends upon the establishment of the immunity. The following observations exemplify the difference of medical opinion as to the cause of symptoms in the living. They are taken from the local dispensary and compare the results of two examinations by different officers made independently at a short interval time. They refer to 64 cases taken at random. There was disagreement in 42 cases, and assuming that either might have been right, this would give a total error of 21 per cent., or 10 per cent. in excess and 10 per cent. in defect. If early cases only were considered, then the disagreement amounts to 73 per cent., or a probable error of 36 per cent., that is, 18 per cent. in excess or defect. It is an ominous sidelight on treatment if we admit a 18 per cent. error of excess, for obviously if in this proportion the discomfort of which complaint is made is not dependent on Pulmonary or other variety of Tuberculosis, then a reasonable probability of permanent cure exists. These observations may be crude and fallacious in inference, but they are suggestive. We can at least conclude that notification measures the discomfort that the population is suffering from on account of this parasite and for comparative purposes is of use. It does not and cannot measure the potentially infected individuals.

No comprehensive attempt has been made in this area to test a large section of the apparently healthy population with tuberculin, and to ascertain in what proportion an intolerance has been created by previous infection. From the number of instances where the product has been used for diagnostic purposes, the area does not appear to vary materially from others where this test has been more systematically applied. It would seem as if the statement previously made, that all have been infected by the fourteenth year and have reacted in some way, may be safely applied locally. If such is the case, then little change can take place in prevalence in spite of the variations in the number complaining of discomfort.

Pulmonary Tuberculosis.

| | 0-1 yrs. | 1-5 yrs. | 5-15 yrs. | 15-25 yrs | 25-45 yrs. | 45-65 yrs. | Over 65 yrs. |
|---|---|---|---|---|---|---|---|
| Percentage deaths to number notified ... | 68 | 22·3 | 9·2 | 34·3 | 40 | | 61·9 |

Other Tubercular Diseases.

| | 0-1 yrs. | 1-5 yrs. | 5-15 yrs. | 15-25 yrs. | 25-45 yrs. | 45-65 yrs. | Over 65 yrs. |
|---|---|---|---|---|---|---|---|
| Average number received annually | 2·6 | 10·2 | 21·8 | 8·3 | 7·5 | 3 | 0·5 |
| Average number of deaths............ | 2·8 | 5·3 | 2·95 | 0·615 | 1·3 | | 0·15 |
| Percentage deaths to number notified | 100 | 51·9 | 13·5 | 7·4 | 12·3 | | 30 |

The percentage deaths to the number notified does not measure case mortality, but it does give some indication of the interval of time between onset of discomfort and death. Should this interval vary, then it must be dependent upon either the organism or the host. Thus the interval between the onset of discomfort and death is longest for Phthisis in the 5-15 age period and for other forms of Tuberculosis 15 to 25. That is to say, that once the balance is against the patient, the structural changes leading to death take the most time in the 5-25 age period.

* Quoted from "Tuberculosis, Heredity and Environment." (1912.) Karl Pearson. Also "The Fight against Tuberculosis and the Death-rate from Phthisis." (1911.)

**The Fundamental Biological Factors Underlying the Incidence of Tuberculosis—(*cont.*).**

The whole of the notifications received since 1908 have been followed up and as far as possible their fate ascertained. Systematic visiting did not commence until 1910. (In the early years notification was limited to Poor Law cases only.) Confining attention to this period, 1,040 cases of Pulmonary Tuberculosis have been notified; 23 per cent. of these may be deducted as the estimated error of diagnosis; this leaves approximately 800 cases. A further 62 of these have left the district, reducing the total to 740. Of this a portion have only recently been diagnosed, and allowing three years as the average period of the onset of discomfort to death, another 200 must be deducted; thus the figure is reduced to 500, and of these 220 are known to have died. Hence 280 still survive, most of which are children The material up to the present is hardly sufficient to construct a life table and thereby estimate the mean after-life-time. It does appear as if at least 50 per cent. of those notified, about whom two medical men agree to the diagnosis, will be dead within two to three years.

The total notifications received during the year 1920 was below the average, but the condition unfortunately does not appear to be permanent. The cause as previously given is dependent on the epidemic of influenza, which disease removed a number of people suddenly who should in the ordinary course of events have been notified from this disease in subsequent years. This state seems, at present time of writing, to be passing away.

The age distribution of notification, as will be seen on reference to the table, differs considerably from the age distribution at death.

To fix our attention on the dying consumptive and blame him for all the mischief has always appeared to me to be both unjust and unwarrantable. I am convinced that the segregation of all those showing obvious signs, and where life is likely to be terminable within a few years, would have little effect on the total amount of floating contagion in our midst. This point has been fully dealt with by Newsholme and Pearson. The weight of argument must be said to be with the latter.

Turning to our local distribution, it is easily seen that Tuberculosis is a frequent cause of poverty, and that the disease may be notified from one area and the death recorded in another, the change being necessitated by the impoverished family exchequer. (The migiation of populations and its nature is fully dealt with by Booth and, with special reference to Tuberculosis, by Snow.)

Passing now to some general questions related to "the heredity and infection factors," the following data have been collected locally. In so far as the results found agree closely with observations made elsewhere, the facts may be taken as correct. Taking the incidence of conjugal phthisis first. (These points have been more fully dealt with by Karl Pearson and the present statement shows their local applicability.)

If one mate is consumptive, is the chance of the other becoming similarly infected greater than would be expected in ordinary circumstances? The figures are as follows: It is important that no bias should exist in the selection of husband and wives such as discomfort or physical signs of Pulmonary Tuberculosis amongst a dispensary population. The inclusion of wives, if they have been examined as contacts, spoils the basis of selection. The examiner expects to find signs of disease and succeeds. Some correlation would be expected, as disease in the husband necessarily prejudices the wife as the family income decreases under such conditions.

| | | Husband. | | |
|---|---|---|---|---|
| | | Not Phthisical. | Phthisical. | Total. |
| Wife | Not Phthisical | 1,060 | 92 | 1,152 |
| | Phthisical | 58 | 10 (5·61) | 68 |
| | Total | 1,118 | 102 | 1,220 |

$r = \cdot 21 \pm \cdot 021$

Eye Colour.

| | | Husband. | | |
|---|---|---|---|---|
| | | Not Blue Eyed. | Blue Eyed. | Total |
| Wife | Not Blue Eyed | 90 | 35 | 125 |
| | Blue Eyed | 41 | 42 (30·8) | 83 |
| | Total | 131 | 77 | 208 |

$r = \cdot 35 \pm \cdot 048$

From the Registrar-General's report it is found that the total number of married couples in 1911 (both living) in Barking was 5,741. The total number of deaths from tuberculosis in the dispensary population during the period was 124, and for the whole town the number was 327. That is about $\frac{124}{327}$ of the total phthisis population belongs to the dispensary, but only a little over one-quarter of corresponding ages attend the dispensary; hence total married population under observation may be regarded as 1,220.

Out of 1,220 couples, as a matter of chance, approximately in six instances would both be expected to die of phthisis in five years, the number actually found was ten, a difference of four.

We find, however, that in mating, there is a marked tendency for like to mate with like. In the same population as the above 180 couples should have had blue eyes; as an actual fact 240 were found. If any other character had been taken the same would have been noted. Hence there must be a tendency for the phthisical or those likely to become phthisical to mate.

(*To be concluded*).

---

At the December meeting of the General Nursing Council for Scotland (Sir John Lorne MacLeod, G.B.E., in the chair), it was decided on the recommendation of the Scottish Board of Health, that the fees for Nurses in training before issue of rules should be raised to the same fees as those charged by the General Nursing Council for England and Wales and the General Nursing Council for Ireland, namely, £2 2s. in the case of the first part of the Register, and £1 1s. in the case of any second or subsequent part of the Register. It was decided, however, that such increase should not take effect until March 1, 1922, so that all applications received by February 28 will be accepted at the present scale.

